# Formation and Geological Sequestration of Uranium Nanoparticles in Deep Granitic Aquifer

**DOI:** 10.1038/srep22701

**Published:** 2016-03-07

**Authors:** Yohey Suzuki, Hiroki Mukai, Toyoho Ishimura, Takaomi D. Yokoyama, Shuhei Sakata, Takafumi Hirata, Teruki Iwatsuki, Takashi Mizuno

**Affiliations:** 1 Graduate School of Science, The University of Tokyo, 7-3-1 Hongo Bunkyo-ku, Tokyo 113-0033, Japan; 2 National Institute of Technology, Ibaraki College, 866 Nakane, Hitachinaka-shi, Ibaraki 312-8508, Japan; 3 Division of Earth & Planetary Sciences, Kyoto University, Kitashirakawa Oiwakesho, Sakyo-ku, Kyoto, 606-8502, Japan; 4 Japan Atomic Energy Agency (JAEA), 1-64 Yamanouchi, Akeyo-cho, Mizunami, Gifu 509-6132, Japan

## Abstract

The stimulation of bacterial activities that convert hexavalent uranium, U(VI), to tetravalent uranium, U(IV), appears to be feasible for cost-effective remediation of contaminated aquifers. However, U(VI) reduction typically results in the precipitation of U(IV) particles less than 5 nanometers in diameter, except for environmental conditions enriched with iron. Because these tiny particles are mobile and susceptible to oxidative dissolution after the termination of nutrient injection, *in situ* bioremediation remains to be impractical. Here we show that U(IV) nanoparticles of coffinite (U(SiO_4_)_1−x_(OH)_4x_) formed in fracture-filling calcium carbonate in a granitic aquifer. *In situ* U-Pb isotope dating demonstrates that U(IV) nanoparticles have been sequestered in the calcium carbonate for at least 1 million years. As the microbiologically induced precipitation of calcium carbonate in aquifer systems worldwide is extremely common, we anticipate simultaneous stimulation of microbial activities for precipitation reactions of calcium carbonate and U(IV) nanoparticles, which leads to long-term sequestration of uranium and other radionuclides in contaminated aquifers and deep geological repositories.

Groundwater contamination with uranium is a serious international problem where uranium was mined, enriched and fabricated for nuclear fuels and weapons^1^. Uranium is immobilized in contaminated groundwater by the addition of organic nutrients to stimulate microbial activity during laboratory incubations and at several contaminated sites^2^,^3^,^4^,^5^,^6^,^7^. However, the immobilized form of uranium is prevalently nanoparticles^2^,^7^,^8^,^9^,^10^, which is of concern because nanoparticles are transported in groundwater as colloids and are easily re-oxidized after the cessation of nutrient amendment^8^,^11^. For the bioremediation technology to be practical, the long-term immobilization of uranium nanoparticles is necessary. Uranium is enzymatically reduced by many species of anaerobic bacteria^11^,^12^, and anaerobic bacteria generally induce calcium carbonate precipitation by increasing aqueous carbonate species from the oxidation of organic matter^13^. As calcium carbonate minerals are stable over geological time scales^14^,^15^, the stimulation of heterotrophic activities in anaerobic aquifers coupled to enzymatic uranium reduction might result in the *in situ* removal of uranium nanoparticles embedded in calcium carbonate. To demonstrate a new form of uranium immobilization in nature, we investigated fracture-filling calcium carbonate formed in a sulfate-reducing granitic aquifer at the vicinity of a uranium ore deposit^16^,^17^.

## Results and Discussions

**Figure 1:**
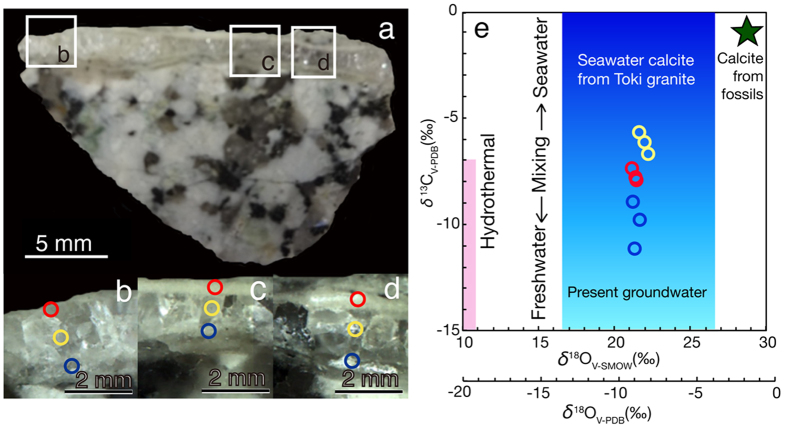
Photographs of a section of granite matrix with calcium carbonate layers where micromilling was conducted from three different spots and a diagram showing the *δ*^13^C and *δ*^18^O values of calcium carbonate from the Tono area. (**a**) Whole sample image, letters within rectangles correspond to (**b–d**). (**b**–**d**) Higher magnification images highlighted with red, yellow and blue circles showing locations for micromilling. (**e**) Plots of *δ*^13^C and *δ*^18^O values of micromilled calcium carbonate from each point in (**b–d**). The ranges of *δ*^13^C and *δ*^18^O values are also shown for calcium carbonate precipitated from hydrothermal fluids and seawater in the Tono area^13^. *δ*^13^C and *δ*^18^O values of calcium carbonate precipitated from the present groundwater were calculated from previously published geochemical data^16^ and fractionation factors^29^.

**Figure 2:**
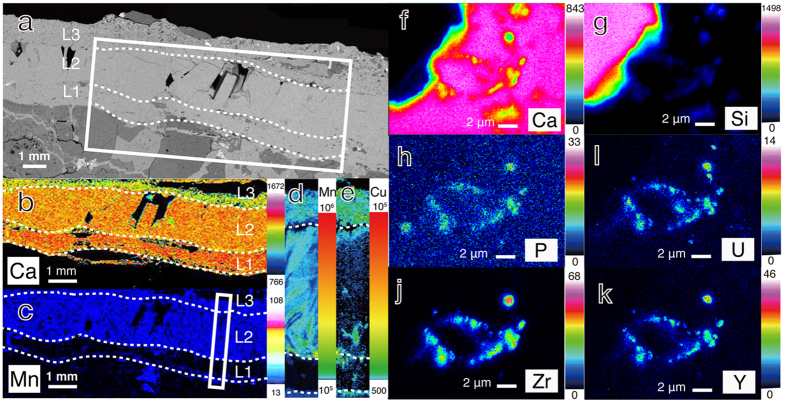
Elemental distributions of calcium carbonate layers. (**a**) Back-scattering electron image with a rectangle highlighted for elemental mapping with EPMA for Ca (**b**) and Mn (**c**). LA-ICP-MS imaging of ^55^Mn (**d**) and ^65^Cu (**e**) from an area highlighted with a rectangle in Fig. 2c. High-resolution FE-EPMA elemental mapping of the calcium carbonate layer (L3) with uranium-bearing loci for Ca (**f**), Si (**g**), P (**h**), U (**i**), Zr (**j**) and Y (**k**). Levels of characteristic X-rays and signal intensity (cps) are expressed in colour spectra for EPMA and LA-CIP-MS, respectively.

A well-developed fracture-filling vein of calcium carbonate in granite was collected from a 200-m deep horizontal borehole (07MI07) at the Mizunami Underground Laboratory (MIU) in Japan. The MIU is located 3 km apart from the main orebody of Tono uranium deposits^18^, and the iron content is low in groundwater^16^,^17^. The vein has three visibly distinct layers: a transparent, well crystalline layer is interleaved with translucent, thin layers (Fig. 1a–d). To elucidate the source of groundwater from which each calcium carbonate layer precipitated, powder samples were collected by micromilling^19^, and then stable isotopic compositions (*δ*^13^C and *δ*^18^O) of sub-microgram quantities of the calcium carbonate were determined by continuous-flow isotope ratio mass spectrometry (CF-IRMS)^20^. Distinct *δ*^13^C and *δ*^18^O values of the three layers ranged between those precipitated from seawater and the present groundwater (Fig. 1e, Supplementary Table 1, Supplementary Note 1). As the study area was submerged in seawater during the Miocene marine transgression (18–15 Ma)^15^, the calcium carbonate layers are inferred to have formed by variable groundwater mixing between seawater and freshwater across the transgression event. To confirm whether the calcium carbonate layers have distinct formation stages, electron probe microanalysis (EPMA) and laser ablation inductively coupled plasma mass spectrometry (LA-ICP-MS) were used for elemental mapping of a polished section (Fig. 2; Supplementary Table 2). As the distributions of Ca, Mn and Cu were clearly distinct in the layers (Fig. 2b–e; hereafter referred to as L1-3), it is clear that each layer had a different formation stage.

**Figure 3:**
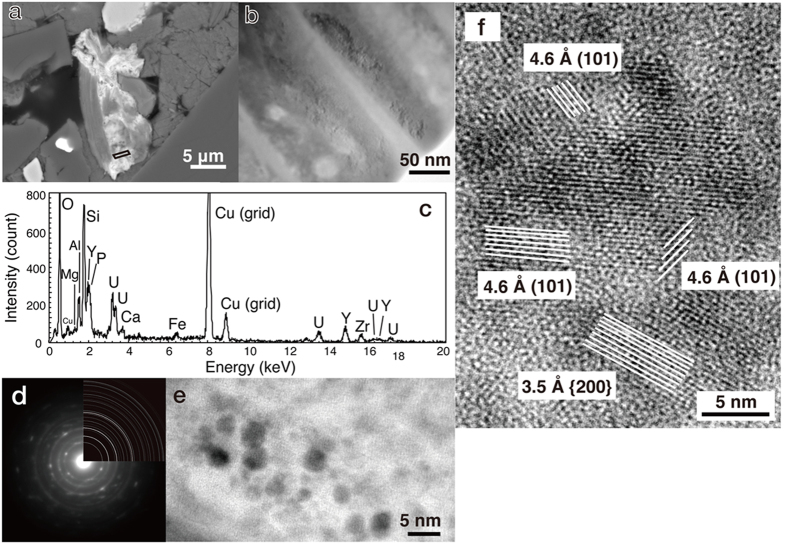
Crystallographic characterizations of U-bearing loci in the calcium carbonate layer (L3) associated with Mg- and Fe-bearing aluminosilicate. (**a**) Back-scattered electron image highlighted with a parallelogram for specimen preparation by the FIB fabrication. (**b**) Low-magnification TEM image of Mg- and Fe-bearing aluminosilicate with U-bearing particles. (**c**) EDS spectrum of Mg- and Fe-bearing aluminosilicate with U-bearing particles. (**d**) SAED pattern from a region enriched with U-bearing particles and that calculated from the coffinite structure (upper right). (**e**) TEM image of individual U-bearing nanoparticles. (**f**) High-resolution TEM image of randomly oriented coffinite nanoparticles showing fringe spacings of 3.5 and 4.6 Å, which correspond to *d*_200_ and *d*_101_ of coffinite.

High-resolution elemental mapping of the L3 layer with field emission electron probe microanalysis (FE-EMPA) further revealed that U-bearing loci were embedded in calcium carbonate and distributed with Si, P, Zr and Y (Fig. 2f–k). To clarify nm-scale characteristics of the U-bearing loci, a focused ion beam (FIB) was used to fabricate ultrathin specimens for transmission electron microscopy (TEM) coupled to energy dispersive x-ray spectroscopy (EDS; Fig. 3). U-bearing loci associated with Mg- and Fe-bearing aluminosilicate were observed to be the aggregates of nanoparticles with dark contrast (Figs. 3a–b). EDS analysis revealed that U-bearing nanoparticles are composed of O, Si, P, Zr and Y (Fig. 3c). Selected area electron diffraction (SAED) pattern analysis also revealed the presence of polycrystalline nanoparticles with a crystal structure identical to coffinite (U(SiO_4_)_1−x_(OH)_4x_; Fig. 3d). Coffinite is mainly composed of U(IV) and isostructural with zircon (ZrSiO_4_) and xenotime (YPO_4_) substituted with Zr and Y for the U site and P for the Si site, respectively^21^. High-resolution TEM observations further confirmed that exceedingly small particles with a size range of <5 nm in diameter are crystalline with lattice fringe spacings of 3.5 Å and 4.6 Å attributed to the *d*_200_ and *d*_101_ of coffinite, and that the coffinite nanoparticles are randomly oriented within small areas (Figs. 3e–f).

One process that is well known to increase uranium mobility in deep granitic bedrocks is the infiltration of oxidizing shallow groundwater^22^. This process is unlikely, because the buffering capacity attributed to microbial sulfate reduction appears to maintain reducing conditions in the present groundwater^17^. Additionally, the rims of pyrite grains were not oxidized to form Fe(III) oxides (Supplementary Fig. 1), which is common in granitic bedrocks intruded with oxidizing groundwater^23^. The intrusion of oxidizing groundwater causes the acidification of groundwater via oxidative pyrite dissolution^22^, the lack of which might be important for the long-term stability of calcium carbonate. The other major process to mobilize uranium is the formation of U(VI)-carbonate complexes^22^, which is likely because the precipitation of calcium carbonate is favorable in groundwater with high concentrations of aqueous carbonate species. To our knowledge, the confinement of coffinite nanoparticles within fracture-filling calcium carbonate is a novel form of uranium immobilization in nature. The formation of calcium carbonate is inferred to be induced by the oxidation of organic matter to dissolved inorganic carbon by anaerobic microbes known to oversaturate groundwater with ^13^C-depleted calcium carbonate^13^. For the precipitation of coffinite nanoparticles, it is likely that U(VI) complexes with carbonate were enzymatically reduced by heterotrophic microorganisms^11^,^12^,^24^, because coffinite nanoparticles were distributed in the calcium carbonate layer without mineral phases such as Fe(III)-bearing minerals and iron sulfides (Fig. 2), which are known to inorganically catalyze U(VI) reduction and/or to adsorb U(IV)^7^,^25^,^26^,^27^,^28^,^29^,^30^,^31^. In addition, the embedment of coffinite nanoparticles in calcium carbonate is thought to prevent the dispersal of uranium in groundwater as colloids.

To estimate the duration of coffinite nanoparticles sequestered in calcium carbonate, the age of coffinite needs to be determined. The L3 layer is inferred to have formed after the Miocene marine transgression event, which can be clarified by applying ^238^U-^206^Pb and ^235^U-^207^Pb isotope series to determine formation age^22^. However, layer-specific micromilling and subsequent digestion for bulk isotope analysis is not applicable, because PbS is present as a minor component in the L3 layer (Supplementary Fig. 1). Instead, a 2-μm sized ArF excimer laser was used to ablate each U-bearing locus embedded in calcium carbonate for isotope measurements of ^235^U, ^207^Pb, ^206^Pb, ^204^(Pb+Hg) and ^202^Hg by multi-collector (MC-) ICP-MS, as previously developed for young zircon^32^. By conservatively taking matrix effects and contributions from co-occurring calcium carbonate into account, the formation age of coffinite was estimated to be 3.7 ± 2.8 Ma or 2.3 ± 0.4 Ma (Supplementary Tables 3–4, Supplementary Fig. 2, Supplementary Note 2). Although these formation ages are younger than expected, it is demonstrated that coffinite nanoparticles have been concealed in calcium carbonate for at least 0.9 million years.

U(IV)-bearing nanoparticles have been preserved in Precambrian sedimentary rocks enriched with solid organic matter^33^,^34^. However, this geological sequestration of U(IV)-bearing nanoparticles is uncontrolled by humans and not relevant to contaminated settings. For the removal of radioactive strontium (^90^Sr) from groundwater, laboratory and field-scale tests have successfully stimulated microbial ureolysis for the formation of calcium carbonate by the amendment of urea^35^,^36^. The integration and fine-tuning of the two independently developed *in situ* strategies has great potential to control the dispersal of uranium and other long-lived radionuclides in the near-surface environment and deep geological formations for the long-term isolation required to reduce radiotoxicity^22^.

## Methods

### Stable carbon and oxygen isotope analysis of calcium carbonate

The granite matrix and calcium carbonate fill was mounted in epoxy and cut into 1-mm-thick sections. The sections were polished and each calcium carbonate layer was micromilled using the Geomill 326 computer-controlled system (Izumo Web Co. Ltd., Shimane, Japan)^19^. 0.2 to 6.7 μg of micromilled calcium carbonate was reacted with phosphoric acid (H_3_PO_4_) in reaction tubes. The evolved CO_2_ was purified and introduced into an IsoPrime100 isotope ratio mass spectrometer (Stockport, United Kingdom) using the continuous-flow gas preparation system (MICAL3c)^20^,^37^. The external precision of this system is better than ± 0.10% for both *δ*^13^C and *δ*^18^O. All data are reported in standard δ notations (*δ*^13^C and *δ*^18^O; %) relative to Vienna–Pee Dee belemnite (V-PDB) and Vienna standard mean ocean water (V-SMOW).

### Scanning electron microscopy (SEM) and electron probe microanalysis (EPMA)

The polished section was coated with carbon and observed by SEM using a JEOL JSM-7000 F instrument (Tokyo, Japan) equipped with energy dispersive X-ray spectroscopy (EDS) at an accelerating voltage of 15 kV. For EPMA, the polished section was coated with carbon, and a JEOL JXA-8900 L instrument at an accelerating voltage of 15 kV was used to obtain elemental mapping. For high-resolution elemental mapping by EPMA, a JEOL JSM-7000 F instrument equipped with a field emission (FE) electron gun was operated at an accelerating voltage of 15 kV.

### Focused ion milling (FIB) fabrication and transmission electron microscopy (TEM)

To observe uranium-bearing loci by TEM, we fabricated electron-transparent ultrathin sections with a focused ion beam (FIB) sample preparation technique using a Hitachi FB-2100 instrument (Ibaraki, Japan) with a micro-sampling system. Before FIB fabrication, the thin section sample was coated by a carbon film and inserted into the FIB apparatus, then locally coated with the deposition of W (100–500 nm thick) for protection, trimmed using a Ga ion beam at an accelerating voltage of 30 kV and thinned down to a final thickness of 100–200 nm with a low energy beam of 10 kV as a final process. FIB-fabricated ultrathin sections were placed on a Cu specimen support with W deposition and observed by TEM. TEM examinations were performed at an accelerating voltage of 200 kV using a JEOL JEM-2010 UHR (LaB_6_ electron gun) with a nominal point resolution of ~0.2 nm. TEM-EDS was used for elemental analysis of U-bearing nanoparticles and Mg- and Fe-bearing aluminosilicate.

### LA-ICP-MS and LA-MC-ICP-MS

An ESI-New Wave Research NWR193 laser ablation system (Fremont, CA, USA) was used for elemental mapping and coffinite dating (see Supplementary Table 2 for instrumental and operational conditions). A Thermo Fisher Scientific iCAP Q quadrupole ICP-MS (Bremen, Germany) was used for elemental mapping, while a Nu instruments Nu Plasma II high resolution MC-ICP-MS (Wrexham, UK) was used for the dating of coffinite. Owing to the non-radiogenic Pb from the surrounding calcium carbonate, the measured Pb/U ratios are plotted discordantly in a concordia plot (Supplementary Fig. 2). Plotting and age calculations were performed using Isoplot 4.15 software^38^. All errors are provided at the 2σ level of uncertainty.

## Additional Information

**How to cite this article**: Suzuki, Y. *et al.* Formation and Geological Sequestration of Uranium Nanoparticles in Deep Granitic Aquifer. *Sci. Rep.* **6**, 22701; doi: 10.1038/srep22701 (2016).

## Supplementary Material

Supplementary Information

## References

[b1] Energy, U. S. D. o. Linking legacies: connecting the cold war nuclear weapons production processes to their environmental consequences. (US Department of Energy, Office of Environmental Management, 1997).

[b2] Newsome L., Morris K., Shaw S., Trivedi D. & Lloyd J. R. The stability of microbially reduced U (IV); impact of residual electron donor and sediment ageing. Chem. Geol. 409, 125–135 (2015).

[b3] Long P. E. *et al.* Bicarbonate impact on U (VI) bioreduction in a shallow alluvial aquifer. Geochim. Cosmochim. Acta 150, 106–124 (2015).

[b4] Istok J. *et al.* *In situ* bioreduction of technetium and uranium in a nitrate-contaminated aquifer. Environ.Sci. Technol. 38, 468–475 (2004). 14750721 10.1021/es034639p

[b5] Anderson R. T. *et al.* Stimulating the *in situ* activity of Geobacter species to remove uranium from the groundwater of a uranium-contaminated aquifer. Appl. Environ. Microbiol. 69, 5884–5891 (2003). 14532040 10.1128/AEM.69.10.5884-5891.2003 PMC201226

[b6] Campbell K. M. *et al.* Geochemical, mineralogical and microbiological characteristics of sediment from a naturally reduced zone in a uranium-contaminated aquifer. Appl. Geochem. 27, 1499–1511 (2012).

[b7] Bargar J. R. *et al.* Uranium redox transition pathways in acetate-amended sediments. Proc. Nat. Acad. Sci. USA. 110, 4506–4511 (2013).

[b8] Suzuki Y., Kelly S. D., Kemner K. M. & Banfield J. F. Radionuclide contamination: Nanometre-size products of uranium bioreduction. Nature 419, 134 (2002). 12226656 10.1038/419134a

[b9] Suzuki Y., Kelly S. D., Kemner K. M. & Banfield J. F. Microbial populations stimulated for hexavalent uranium reduction in uranium mine sediment. Appl. Environ. Microbiol. 69, 1337–1346 (2003). 12620814 10.1128/AEM.69.3.1337-1346.2003 PMC150047

[b10] Bernier-Latmani R. *et al.* Non-uraninite products of microbial U (VI) reduction. Environ. Sci. Technol. 44, 9456–9462 (2010). 21069950 10.1021/es101675a

[b11] Newsome L., Morris K. & Lloyd J. R. The biogeochemistry and bioremediation of uranium and other priority radionuclides. Chem. Geol. 363, 164–184 (2014).

[b12] Suzuki Y. & Suko T. Geomicrobiological factors that control uranium mobility in the environment: Update on recent advances in the bioremediation of uranium-contaminated sites. J. Mineral. Petrol. Sci. 101, 299–307 (2006).

[b13] Drever J. I. The geochemistry of natural waters: surface and groundwater environments. (Prentice Hall, 1997).

[b14] Milodowski A. *et al.* Application of mineralogical, petrological and geochemical tools for evaluating the palaeohdrogeological evolution of the PADAMOT study sites. ADAMOT Project Technical Report WP2 206UK Nirex Ltd, Harwell (2005).

[b15] Iwatsuki T., Satake H., Metcalfe R., Yoshida H. & Hama K. Isotopic and morphological features of fracture calcite from granitic rocks of the Tono area, Japan: a promising palaeohydrogeological tool. Appl. Geochem. 17, 1241–1257 (2002).

[b16] Iwatsuki T., Hagiwara H., Ohmori K., Munemoto T. & Onoe H. Hydrochemical disturbances measured in groundwater during the construction and operation of a large-scale underground facility in deep crystalline rock in Japan. Environmental Earth Sciences, 1–17 (2015).

[b17] Suzuki Y. *et al.* Biogeochemical Signals from Deep Microbial Life in Terrestrial Crust. PloS one 9, e113063 (2014). 25517230 10.1371/journal.pone.0113063 PMC4269445

[b18] Sasao E. *et al.* An overview of a natural analogue study of the Tono Uranium Deposit, central Japan. Geochem. Explor. Environ. A. 6, 5–12 (2006).

[b19] Sakai S. & Kodan T. Micropowder collecting technique for stable isotope analysis of carbonates. Rapid Commun. Mass Spectrom. 25, 1205–1208 (2011). 21488119 10.1002/rcm.4980

[b20] Ishimura T., Tsunogai U. & Gamo T. Stable carbon and oxygen isotopic determination of sub‐microgram quantities of CaCO_3_ to analyze individual foraminiferal shells. Rapid Commun. Mass Spectrom. 18, 2883–2888 (2004). 15517527 10.1002/rcm.1701

[b21] Deditius A. P., Pointeau V., Zhang J. M. & Ewing R. C. Formation of nanoscale Th-coffinite. Am. Mineral. 97, 681–693 (2012).

[b22] Langmuir D. Aqueous environmental geochemistry. (Upper Saddle River, NJ: Prentice Hall, 1997).

[b23] Drake H., Tullborg E.-L. & MacKenzie A. B. Detecting the near-surface redox front in crystalline bedrock using fracture mineral distribution, geochemistry and U-series disequilibrium. Appl. Geochem. 24, 1023–1039 (2009).

[b24] Cai C. F. *et al.* Mineralogical and geochemical evidence for coupled bacterial uranium mineralization and hydrocarbon oxidation in the Shashagetai deposit, NW China. Chem. Geol. 236, 167–179 (2007).

[b25] Latta D. E., Boyanov M. I., Kemner K. M., O’Loughlin E. J. & Scherer M. M. Abiotic reduction of uranium by Fe (II) in soil. Appl. Geochem. 27, 1512–1524 (2012).

[b26] Du X. *et al.* Reduction of uranium (VI) by soluble iron (II) conforms with thermodynamic predictions. Environ. Sci. Technol. 45, 4718–4725 (2011). 21553877 10.1021/es2006012

[b27] Hyun S. P., Davis J. A., Sun K. & Hayes K. F. Uranium (VI) reduction by iron (II) monosulfide mackinawite. Environ. Sci. Technol. 46, 3369–3376 (2012). 22316012 10.1021/es203786p

[b28] Kelly S. D. *et al.* Speciation of uranium in sediments before and after *in situ* biostimulation. Environ. Sci. Technol. 42, 1558–1564 (2008). 18441803 10.1021/es071764i

[b29] Kelly S. D. *et al.* Uranium transformations in static microcosms. Environ. Sci. Technol. 44, 236–242 (2010). 19958005 10.1021/es902191s

[b30] Watson D. B. *et al.* *In situ* bioremediation of uranium with emulsified vegetable oil as the electron donor. Environ Sci. Technol. 47, 6440–6448 (2013). 23697787 10.1021/es3033555

[b31] Wu W.-M. *et al.* Effects of nitrate on the stability of uranium in a bioreduced region of the subsurface. Environ. Sci. Technol. 44, 5104–5111 (2010). 20527772 10.1021/es1000837

[b32] Sakata S. *et al.* Determination of U–Pb Ages for Young Zircons using Laser Ablation‐ICP‐Mass Spectrometry Coupled with an Ion Detection Attenuator Device. Geostand. Geoanal. Res. 38, 409–420 (2014).

[b33] Fuchs S., Schumann D., Williams-Jones A. & Vali H. The growth and concentration of uranium and titanium minerals in hydrocarbons of the Carbon Leader Reef, Witwatersrand Supergroup, South Africa. Chem. Geol. 393, 55–66 (2015).

[b34] Fayek M., Utsunomiya S., Ewing R. C., Riciputi L. R. & Jensen K. A. Oxygen isotopic composition of nano-scale uraninite at the Oklo-Okélobondo natural fission reactors, Gabon. Am. Mineral. 88, 1583–1590 (2003).

[b35] Lauchnor E. G. *et al.* Bacterially induced calcium carbonate precipitation and strontium coprecipitation in a porous media flow system. Environ. Sci. Technol. 47, 1557–1564 (2013). 23282003 10.1021/es304240y

[b36] Fujita Y. *et al.* Stimulation of microbial urea hydrolysis in groundwater to enhance calcite precipitation. Environm. Sci. Technol. 42, 3025–3032 (2008). 10.1021/es702643g 18497161

[b37] Ishimura T., Tsunogai U. & Nakagawa F. Grain-scale heterogeneities in the stable carbon and oxygen isotopic compositions of the international standard calcite materials (NBS 19, NBS 18, IAEA-CO-1, and IAEA-CO-8). Rapid Communications in Mass Spectrometry 22, 1925–1932 (2008). 18484681 10.1002/rcm.3571

[b38] Isoplot/Ex, A geochronological toolkit for Microsoft Excel, Special Publication, 1a (Berkeley Geochronological Center, Berkeley, CA, 2001).

