# Supplementary material for: Formation and Geological Sequestration of Uranium Nanoparticles in Deep Granitic Aquifer
Source: Sci Rep. 2016 Mar 7;6:22701. doi: 10.1038/srep22701 (PMC4780221; doi:10.1038/srep22701)
Supplement: Supplementary Information [file srep22701-s1.docx]

**Formation and Geological Sequestration of Uranium Nanoparticles in Deep Granitic Aquifer**

Yohey Suzuki^1*^, Hiroki Mukai^1^, Toyoho Ishimura^2^, Takaomi D. Yokoyama^1^, Shuhei Sakata^3^, Takafumi Hirata^3^, Teruki Iwatsuki^4^, Takashi Mizuno^4^

^1^Graduate School of Science, The University of Tokyo, 7-3-1 Hongo Bunkyo-ku, Tokyo 113-0033, Japan

^2^National Institute of Technology, Ibaraki College, 866 Nakane, Hitachinaka-shi, Ibaraki 312-8508, Japan

^3^Division of Earth & Planetary Sciences, Kyoto University, Kitashirakawa Oiwakesho, Sakyo-ku, Kyoto, 606-8502, Japan

^4^Japan Atomic Energy Agency (JAEA), 1-64 Yamanouchi, Akeyo-cho, Mizunami, Gifu 509-6132, Japan

*To whom correspondence should be addressed at the University of Tokyo, 7-3-1 Hongo Bunkyo-ku, Tokyo 113-0033, Japan. E-mail: yohey-suzuki@eps.s.u-tokyo.ac.jp

Supplementary Table 1. Stable carbon and oxygen isotopic compositions of micromilled samples from the calcium carbonate layers with analytical quantities.

|  | δ^13^C  V-PDB | δ^18^O  V-PDB | δ^18^O V-SMOW | CaCO_3_ |
| --- | --- | --- | --- | --- |
|  | (‰) | (‰) | (‰) | weight (μg) |
|  | -8.87 | -9.51 | 21.05 | 6.7 |
| L1 | -11.16 | -9.33 | 21.24 | 5.5 |
|  | -9.81 | -8.99 | 21.59 | 0.8 |
|  | -6.68 | -8.48 | 22.12 | 5.6 |
| L2 | -5.72 | -9.12 | 21.46 | 3.8 |
|  | -6.09 | -8.76 | 21.83 | 5.0 |
|  | -7.86 | -9.29 | 21.29 | 4.7 |
| L3 | -7.80 | -9.26 | 21.31 | 2.9 |
|  | -7.32 | -9.53 | 21.04 | 0.2 |

Supplementary Table 2. Analytical conditions for the in-situ elemental mapping.

| **Laser ablation system** | | |
| --- | --- | --- |
|  | Instrument | NWR193 excimer laser (New Wave Research, Fremont USA) |
|  | Cell type | Two volume cell |
|  | Laser wave length | 193 nm |
|  | Pulse duration | <5 ns |
|  | Fluence | 3.6 J/cm^2^ |
|  | Repetition rate | 20 Hz |
|  | Ablation pit size | 10 μm |
|  | Sampling mode | Line scan |
|  | Pre-cleaning | not made |
|  | Carrier gas | He gas and Ar make-up gas combined outside ablation cell |
|  | He gas flow rate | 0.50 l/min |
|  | Ar make-up gas flow rate | 0.83 l/min |
|  | Signal smoothing device | Not used |
|  |  |  |
| **ICP Mass Spectrometer** | | |
|  | Instrument | iCAP Qc ICP-QMS (Thermo Scientific, Bremen, Germany) |
|  | RF power | 1400 W |
|  | Data reduction | Time resolved analysis |
|  | Detection mode | Pulse counting mode and analog mode |
|  | Monitored isotopes | ^7^Li, ^11^B, ^27^Al, ^48^Ti, ^51^V, ^52^Cr, ^55^Mn, ^57^Fe, ^59^Co, ^60^Ni, ^63^Cu, ^66^Zn, ^75^As, ^77^Se, ^79^Br, ^88^Sr, ^89^Y, ^90^Zr, ^95^Mo, ^125^Te, ^127^I, ^133^Cs, ^137^Ba, ^139^La, ^140^Ce, ^146^Nd, ^182^W, ^208^Pb, ^232^Th, ^238^U |
|  | Integration time per peak | 0.02 s for ^48^Ti, ^55^Mn, ^57^Fe, ^63^Cu and 0.01 s for other isotopes |
|  | Total integration time per reading | 0.383 seconds |
|  | Formation rate of ^232^Th^16^O | <2.5% |
|  |  |  |
| **Conditions for isotope mapping and data processing** | | |
|  | Speed of line scan | 20 μm/s |
|  | Number of lines | 100 lines |
|  | Line spacing | 10 μm |
|  | Interval of each line | 30 seconds |
|  | Gas blank | Gas blank counts were obtained for 10 seconds between line scans. |
|  | Data processing software used for creating image | iQuant2 developed by Dr. Toshihiro Suzuki (Tokyo Institute of Technology) |

Supplementary Table 3. Analytical conditions for in-situ LA-ICP-MS U-Pb dating.

| **Laser ablation system** | | |  |
| --- | --- | --- | --- |
|  | Instrument | NWR193 excimer laser (New Wave Research, Fremont USA) | |
|  | Cell type | Two volume cell | |
|  | Laser wave length | 193 nm | |
|  | Pulse duration | <5 ns | |
|  | Fluence | 7.0 J/cm^2^ | |
|  | Repetition rate | 5 Hz | |
|  | Ablation pit size | 2 μm | |
|  | Sampling mode | Single hole drilling | |
|  | Pre-cleaning | 1 shot with 35-75 μm | |
|  | Carrier gas | He gas and Ar make-up gas combined outside ablation cell | |
|  | He gas flow rate | 0.50 l/min | |
|  | Ar make-up gas flow rate | 0.95 l/min | |
|  | Ablation duration | 20 seconds | |
|  | Signal smoothing device | Enabled | |
|  |  |  | |
| **ICP Mass Spectrometer** | | |  |
|  | Instrument | Nu PlasmaII HR-MC-ICP-MS (Nu Instruments, Wrexham, U.K.) | |
|  | RF power | 1300 W | |
|  | Data reduction | Integration of total ion counts per single ablation. Signals obtaind from first few seconds were not used for data reduction, and next signals obtained from　5.4 seconds were integrated for further calculations. Intensity of ^238^U is calculated assuming ^238^U/^235^U = 137.88 (ref^1^). | |
|  | Detection mode | Multiple collector mode | |
|  | Monitored isotopes | ^202^Hg, ^204^(Hg + Pb), ^206^Pb, ^207^Pb, ^208^Pb, ^232^Th, ^235^U | |
|  | Integration time per peak | 5.4 seconds | |
|  | Total integration time per reading | 0.4 seconds | |
|  | Formation rate of ^232^Th^16^O | <0.4% | |
|  |  |  | |
| **Data processing** | |  | |
|  | Gas blank | Gas blank counts were obtained for 20 s prior to each ablation pit. | |
|  | Calibration strategy | 91500 zircon was used in correction for Pb/U fractionation in all measurements. NIST SRM 610 was used for correction of Pb/Pb fractionation. To estimate the matrix effect between coffinite and zircon standard, the difference between measured ratio and true ratio in ^206^Pb/^238^U for 91500 and NIST SRM 610 was calculated, and the margin of two values was propagated in the final uncertainties. | |
|  | Normalization values | ^206^Pb/^238^U = 0.1792, U concentration = 81.2 μg/g, Th concentration = 28.6 μg/g, Pb concentration = 14.8 μg/g (91500, ref^2^), ^207^Pb/^206^Pb = 0.9096, ^206^Pb/^204^Pb = 17.045, ^207^Pb/^204^Pb = 15.504, ^208^Pb/^204^Pb = 36.964 for NIST SRM 610 (ref^3^). | |
|  | Common-Pb correction | Concordia intercept age was used(ref^4^). | |
|  | Uncertainties | Uncertainties for ages and isotope ratios are quoted at 2 SD absolute, propagation is by quadratic addition. Repeatability of primary standard, counting statistics of measured isotope and the esitimated magnitude of matrix effect are propagated. | |

Supplementary Table 4. Collector configurations used on the Nu Plasma II for the U-Pb isotope analysis.

| Detector^a^ | IC5 | H10 | H9 | H8 | H7 | H6 | H5 | H4 | H3 | H2 | H1 | Ax | L1 | L2 | L3 | L4 | L5 | IC0 | IC1 | D2 | IC3 | IC4 |
| --- | --- | --- | --- | --- | --- | --- | --- | --- | --- | --- | --- | --- | --- | --- | --- | --- | --- | --- | --- | --- | --- | --- |
| Amu | 235 |  | 232 |  |  |  |  |  |  |  |  |  |  |  |  |  |  | 208 | 207 | 206 | 204 | 202 |
| Isotopes | U |  | Th |  |  |  |  |  |  |  |  |  |  |  |  |  |  | Pb | Pb | Pb | Pb, Hg | Hg |

Note the gaps in the collector assembly between H10 and H9, H9 and H8, D2 and IC3, and IC3 and IC4.

^a^H10 to H1, Ax, and L1 to L5 are faraday cups, IC0 to IC5 are secondary electron multipliers, and D2 is a daly cup.

Supplementary Figure 1.

Elemental compositions of particles associated with uranium-bearing loci in the calcium carbonate layer (also shown in Fig. 3). EDS spectrum from a Pb- and S-bearing particle and Na- and K-bearing aluminosilicate particles (upper). EDS spectrum of an Fe- and S-bearing particle (lower).

Supplementary Figure 2. Concordia diagrams of coffinite U-Pb dating. Solid line is concordia line. Dashed line is discordia line. Grey circles are measured Pb/U ratios of coffinite.


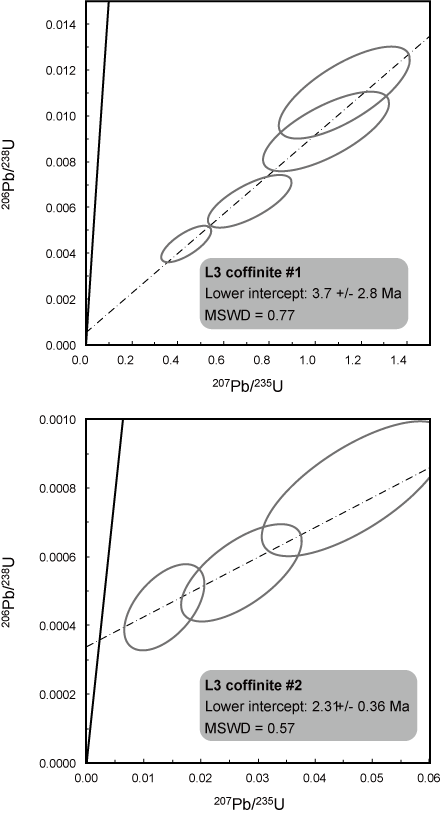


Supplementary Note 1

As shown in Figure 1e and Supplementary Table 1, the translucent layer adjacent to the granite matrix (L1) was the most depleted in ^13^C (−11.16 to −8.87‰ V-PDB) and ^18^O (21.05 to 21.59‰ V-SMOW), whereas the middle crystalline layer (L2) was the most enriched with ^13^C (−6.68 to −5.72‰ V-PDB) and ^18^O (21.46 to 22.12‰ V-SMOW). Another translucent layer (L3) has distinct signatures of *δ*^13^C (−7.86 to −7.32‰ V-PDB) and *δ*^18^O (21.04 to 21.31‰ V-SMOW) that are close to those from the middle crystalline layer. The *δ*^13^C and *δ*^18^O values of calcium carbonate from the middle layer L2 are close to those inferred to have precipitated from seawater[^5^](#_ENREF_1). Regarding calcium carbonate precipitating from the present groundwater of meteoric origin (*δ*^18^O = −9 to −8.8‰ V-SMOW)[^6^](#_ENREF_2), the *δ*^18^O values are calculated to be 22.3 to 22.5‰ V-SMOW using a fractionation factor (α) of 1.0318 at an *in-situ* temperature of 10°C^7^. As the fractionation factor between calcium carbonate and DIC (α = 1.0015) is negligible at 10°C[^8^](#_ENREF_4), calcium carbonate precipitated from the present groundwater has *δ*13C values ranging from −12.4 to −15.8‰ V-PDB[^6^](#_ENREF_1). This excludes the possibility that the calcium carbonate layers have recently precipitated from groundwater. This inference is also supported by the slightly undersaturated state of the present groundwater with respect to calcium carbonate^6^.

Supplementary Note 2

Helium was used as the carrier gas, which further improves transport efficiency with the ICP and also reduces aerosol deposition on the sample surface[^9^](#_ENREF_2). For the dating of coffinite, the instrument was operated to minimize the production of oxide signals (i.e., ^232^Th^16^O^+^/^232^Th^+^ <0.4%) and the measured instrumental mass bias of the ^206^Pb/^238^U ratio from the expected value for zircon. To subtract contributions from non-radiogenic Pb isotopes, pyrite grains close to coffinite nanoparticles in layer L3 were measured. In this study, a matrix matched standard for coffinte was not applied to U-Pb dating. Alternatively, matrix effects between coffinite and zircon were conservatively incorporated into analytical errors by ablating primary reference materials made of glass (NIST SRM 610) and zircon (91500)[^10^](#_ENREF_2). The secondary reference material Prešovice zircon[^11^](#_ENREF_2) was also used for correction of Pb/U isotope ratios. Common Pb corrections were made using ^204^Pb obtained by subtracting ^204^Hg from a total of 204 counts, whereas ^204^Hg was corrected by the measured ^202^Hg. Coffinite ages were determined using lower intercept age.

Supplementary References

1 Jaffey, A., Flynn, K., Glendenin, L., Bentley, W. t. & Essling, A. Precision measurement of half-lives and specific activities of U 235 and U 238. *Phy. Rev. C* **4**, 1889 (1971).

2 Wiedenbeck, M. *et al.* Three natural zircon standards for U‐Th‐Pb, Lu‐Hf, trace element and REE analyses. *Geostand. Newslett.* **19**, 1-23 (1995).

3 Jochum, K. P. *et al.* MPI‐DING glasses: New geological reference materials for in situ Pb isotope analysis. *Geochem. Geophy. Geosys.* **6** (2005).

4 Isoplot/Ex, A geochronological toolkit for Microsoft Excel, Special Publication, 1a (Berkeley Geochronological Center, Berkeley, CA, 2001).

5 Iwatsuki, T., Satake, H., Metcalfe, R., Yoshida, H. & Hama, K. Isotopic and morphological features of fracture calcite from granitic rocks of the Tono area, Japan: a promising palaeohydrogeological tool. *Appl. Geochem.* **17**, 1241-1257 (2002).

6 Suzuki, Y. *et al.* Biogeochemical Signals from Deep Microbial Life in Terrestrial Crust. *PloS one* **9**, e113063 (2014).

7 O'Neil, J. R., Clayton, R. N. & Mayeda, T. K. Oxygen isotope fractionation in divalent metal carbonates. *J. Chem. Phys.* **51**, 5547–5558 (1969).

8 Emrich, K., Ehhalt, D. & Vogel, J. Carbon isotope fractionation during the precipitation of calcium carbonate. *Earth Planet. Sci. Lett.* **8**, 363-371 (1970).

9 Guillong, M. & Günther, D. Effect of particle size distribution on ICP-induced elemental fractionation in laser ablation-inductively coupled plasma-mass spectrometry. *J. Anal. At. Spectrom.* **17**, 831-837 (2002).

10 Wiedenbeck, M. *et al.* Three natural zircon standards for U‐Th‐Pb, Lu‐Hf, trace element and REE analyses. *Geostand. newslett.* **19**, 1-23 (1995).

11 Sláma, J. *et al.* Plešovice zircon—a new natural reference material for U–Pb and Hf isotopic microanalysis. *Chem. Geol.* **249**, 1-35 (2008).
